# Shedding of nontyphoidal *Salmonella* by asymptomatic convalescing children under 5 years as a risk factor for invasive disease in Mukuru informal settlement in Nairobi, Kenya

**DOI:** 10.1128/jcm.00750-24

**Published:** 2024-10-24

**Authors:** Kelvin Kering, Kariuki Njaanake, Celestine Wairimu, Marianne Mureithi, Collins Kebenei, Georgina Odityo, Michael Mugo, Susan M. Kavai, Cecilia Mbae, Kristin Weber, Michael Pietsch, Tanja Pilz, Oliver Drechsel, Andrea Thürmer, Torsten Semmler, Stephan Fuchs, Sandra Simon, Antje Flieger, Lothar H. Wieler, Samuel Kariuki

**Affiliations:** 1 Centre for Microbiology Research, Kenya Medical Research Institute, Nairobi, Kenya; 2 Department of Medical Microbiology and Immunology, University of Nairobi, Nairobi, Kenya; 3 Robert Koch Institute, Wernigerode, Germany; 4 Robert Koch Institute, Berlin, Germany; 5 Digital Global Public Health, Hasso Plattner Institute, Potsdam, Germany; 6 Drugs for Neglected Diseases Initiative, Nairobi, Kenya; Johns Hopkins University, Baltimore, Maryland, USA

**Keywords:** shedding, carriage, *Salmonella*, children, asymptomatic

## Abstract

Nontyphoidal *Salmonella* (NTS) is a predominant cause of invasive disease in sub-Saharan Africa especially among children under 5 years. Asymptomatic fecal shedding of NTS is hypothesized to contribute to the human-to-human transmission of NTS especially in low-resource settings. However, the role of pathogen shedding in invasive disease is unknown. This study aimed to investigate the prevalence and duration of fecal shedding of NTS among children under 5 years convalescing from invasive NTS disease and among healthy individuals in the community. Children presenting with fever of ≥38°C with or without diarrhea were recruited at four health facilities in Nairobi, between June 2021 and August 2023. Blood and stool samples collected were subjected to culture for the isolation of NTS (*S*. Enteritidis and *S*. Typhimurium). Children with NTS culture-positive samples (index cases) were followed up post-acute disease where household contacts and controls provided stool samples for isolation of NTS. NTS prevalence among the 3,293 individuals recruited was 1.52%. Asymptomatic shedding post-treatment was observed in almost one-third (31%) of the 42 index cases followed up. Of the 13 with intestinal shedding, 7 were shedding NTS of the same sequence type (ST) as the one recovered during acute disease. The longest duration of intestinal shedding was 3 months post-treatment. Of the 241 healthy individuals recruited, 8 had asymptomatic shedding of NTS, and 2 of these were closely related to those recovered from index cases. These findings support the hypothesis of human-to-human transmission of NTS in sub-Saharan Africa highlighting the possible benefit of vaccine introduction.

**IMPORTANCE:**

Asymptomatic fecal shedding of nontyphoidal *Salmonella* (NTS) is hypothesized to contribute to the human-to-human transmission of NTS especially in low-resource settings which could lead to invasive disease among high-risk populations, especially children. Our findings reiterate the hypothesis that human reservoirs could be important in the transmission of nontyphoidal *Salmonella* in sub-Saharan Africa. This underscores the importance of developing infection prevention measures which could include vaccine deployment and improving water, sanitation and hygiene infrastructure.

## INTRODUCTION

Nontyphoidal *Salmonella* (NTS) is an important cause of gastroenteritis globally, with approximately 3.4 million cases being detected annually (1). NTS infection often presents as acute self-limiting gastroenteritis (diarrhogenic NTS, dNTS); however, it can lead to invasive (bloodstream) infection and further advance to meningitis or focal infections (2), referred to as invasive NTS (iNTS). In sub-Saharan Africa, NTS is a leading cause of community-onset bloodstream infections, especially among children under 5 years of age. In 2017, 535,000 cases of iNTS disease that resulted in 77,500 deaths were recorded globally, of which 422,000 cases and 66,500 deaths were in sub-Saharan Africa (3). The most vulnerable populations are children below 5 years in particular the malnourished or with malaria (3). Immunocompromised individuals particularly those with human immunodeficiency virus (HIV) are also at high risk (4, 5). The majority of the NTS disease cases reported in sub-Saharan Africa are attributed to two serotypes: *Salmonella enterica* subspecies *enterica* serovars Typhimurium and Enteritidis (hereafter *S*. Typhimurium and *S*. Enteritidis) (6).

NTS is primarily a zoonotic pathogen and transmission to humans is through consumption of contaminated food (7), in particular meat and dairy products (4, 8). However, in sub-Saharan Africa, the reservoir(s) and route(s) of transmission remain unclear with studies hypothesizing the presence of different reservoirs including human (1, 8–10), environmental, and zoonotic (11). The hypothesis of humans as potential NTS reservoirs is based on evidence of human carriage and fecal shedding of NTS and the high genetic similarity between NTS isolates from iNTS cases and asymptomatic hosts especially in disease-endemic settings (1, 7, 12). Asymptomatic carriage of NTS (enteric NTS, eNTS) is not atypical and can either be temporary carriage (transient or convalescent) or chronic carriage (persistent or permanent) (13, 14). However, there is inconsistency in the duration of carriage and shedding that distinguish between temporary and chronic carriage with some studies using either 3 or 12 months (14) to distinguish between the two periods of carriage. Fecal shedding of NTS can also occur either intermittently or continuously.

Several studies have reported on the carriage or fecal shedding of NTS. A recent retrospective study conducted in Israel reported that at least 2.2% of confirmed NTS cases in Israel resulted in prolonged infection (15). A previous study undertaken in Thailand observed that 7.7% of asymptomatic adults were NTS carriers, although none was shedding the initial serotype (16). In India, *Salmonella* carriage was observed in 1% of school-going children (17). In sub-Saharan Africa, asymptomatic NTS carriage ranges between 1.6% and 6.9% in Kenya (1, 9), 6.2% in Burkina Faso (8), 2.1% and 3.4% in the Democratic Republic of Congo (12, 18), and 1.0% and 2.5% in Senegal and Guinea-Bissau, respectively (19). While asymptomatic carriers may not pose a major risk in developed countries, vulnerable populations with immunosuppressive conditions including HIV, malaria, and malnutrition, in low-resourced countries are at higher risk of developing invasive diseases following exposure to NTS from asymptomatic carriers (20). NTS carriage among fecally incontinent individuals could be a source of invasive and/or diarrhoeal NTS disease within households and the community especially among vulnerable children in disease-endemic settings.

Prolonged fecal shedding of NTS could also result in the sustained presence of NTS in the community and environmental contamination in areas with poor water, sanitation, and hygiene (WASH) infrastructure. The emergence of multidrug-resistant NTS (21, 22) in carriage and shedding, complicates the management of the disease, especially in settings where alternative antibiotics are costly or unavailable. Whereas several studies have reported on the shedding of NTS in Kenya, and sub-Saharan Africa, the duration of shedding remains unknown. Given that prolonged fecal shedding could result in new infections, disease endemicity, and environmental contamination, this study aimed to determine the duration of fecal shedding of NTS (*S*. Typhimurium and *S*. Enteritidis) among children under 5 years post-invasive disease and in healthy individuals from households of the cases and the community.

## MATERIALS AND METHODS

### Study site

The study site was Mukuru informal settlement which is located approximately 15 km east of the Nairobi central business district. Mukuru informal settlement is one of the largest slums in Kenya with an estimated population of 250,000 (23). The densely populated informal settlement is characterized by low-income levels, poor-quality housing structures (corrugated iron huts measuring ca. 10 ft. × 10 ft.), inadequate water supply, poor sanitation infrastructure, and solid-waste management (24–26), which contribute to high rates of transmission of infectious diseases.

### Participant enrollment and sample collection

Participants were enrolled from four outpatient health facilities that serve the population from Mukuru: Medical Missionaries of Mary, Reuben Centre, Mukuru City Council Clinic, and Mama Lucy Hospital. Participants were assessed by clinicians to check if they met the inclusion criteria: (i) children below 5 years of age and (ii) with fever or history of fever (≥38°C) for more than 24 h with or without diarrhea (three or more episodes of loose or watery stool in the preceding 24 h). Parents or guardians whose children were eligible for enrollment were then requested to consent to their children’s participation in the study. The participants were recruited between June 2021 and August 2023 and each was assigned a unique identifier that was used for their identification throughout the study.

About 1–3 mL of blood was collected using a butterfly needle and a syringe and then aseptically transferred into Bactec blood culture bottles (BD BACTEC Peds Plus Medium). Stool samples or rectal swabs (in the absence of whole stool) were also collected. Sterile cotton-tipped swabs were then used to transfer the whole stool into Cary-Blair medium (Oxoid Ltd., Basingstoke, UK). Consequently, all fecal samples (rectal swabs and whole stool) were transported to the laboratory as fecal swab samples in Cary-Blair Tansport Medium and delivered to the Microbiology Laboratory at the Kenya Medical Research Institute (KEMRI) in Nairobi for processing, ca. 4–5 h after collection.

### Laboratory processing of samples

#### Blood sample analysis

Bactec blood culture bottles containing blood were processed as described previously (1, 27). Briefly, the blood culture bottles were incubated in a computerized BACTEC 9050 Blood Culture System (BD, Franklin Lakes, NJ, USA) at 37°C for 24 h. The blood cultures were then subcultured onto MacConkey, blood, and chocolate agar (Oxoid, Basingstoke, UK) and incubated at 37°C for 24 h. The blood culture bottles were incubated for a further 7 days and ultimately all were subcultured regardless of bacterial growth status. Suspected *Salmonella* isolates were subcultured on Mueller-Hinton agar and identified through biochemical tests on API (Analytical Profile Index) 20E strips (Biomerieux, Marcy-l'Étoile, France) and serotyping using commercial antisera (Remel, Thermo Fisher Scientific, MA, USA) based on the Kaufman-White Scheme. The threshold for a positive identification was set at 90% for the API 20E test.

#### Stool sample analysis

Rectal swabs or whole stools were initially enriched in Selenite Fecal broth (Oxoid Ltd., Basingstoke, UK) at 37°C overnight. The overnight broth cultures were then subcultured on MacConkey and Xylose Lysine Deoxycholate (XLD) (Oxoid Ltd., Basingstoke, UK) agar and incubated at 37°C overnight. Suspected *Salmonella* colonies were subsequently subcultured on Mueller-Hinton agar (Oxoid Ltd., Basingstoke, UK) and identified through biochemical tests using API 20E test and serotyping (1).

### Index cases follow-up and enrollment of healthy individuals

Index cases were put on treatment following a clinician’s prescription. Household follow-up of the index cases was done 14 days post-completion of treatment. During follow-up, healthy individuals (contacts) residing in the same households with the index cases were requested to participate in the study after providing an informed written consent. Whole stool samples or rectal swabs were collected from the index cases and their contacts. The age and gender details of the contacts were recorded. As control, 100 m from the case-contact household, a household was randomly selected and healthy children under 5 years (controls), and other household members were recruited after written informed consent. The household contacts and controls were enrolled only if they had not been on antibiotics in the past month or during enrollment and were not experiencing diarrhoeal symptoms. If the selected household did not satisfy the inclusion criteria or declined to participate, an alternative household was selected. Stool or rectal swabs were then collected from the participants in the control households and transported to the laboratory in KEMRI within 4–5 h.

### Longitudinal follow-up

**Fig 1:**
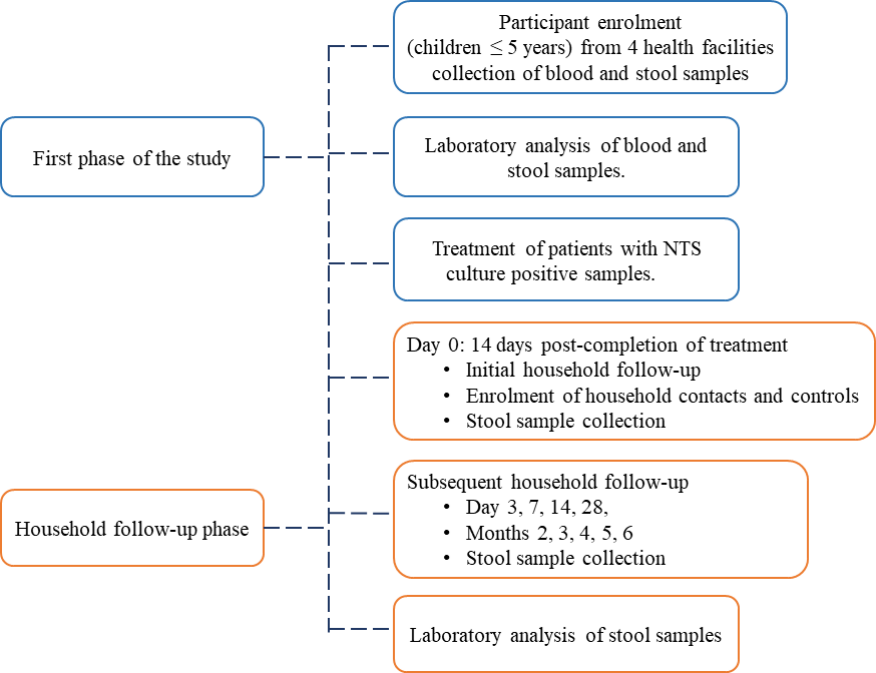
Flowchart indicating participant enrollment during the first phase of the study, and household follow-up. The timeline for household follow-up was: month 1; day 0 (first household follow-up), days 3, 7, 14, 28, and monthly after that. Follow-up was stopped after three consecutive *Salmonella* negative cultures of fecal samples. Household contacts were recruited from the same households as the index cases, while contacts were recruited 100 m away from the case-contact household.

After the initial sampling of the index cases and recruitment of contacts and controls, longitudinal sampling was carried out five times [day 0 (first day of follow-up/14 days post-completion of treatment of index cases), days 3, 7, 14, and 28] in the first month for all index cases and thereafter once every month for individuals with shedding (Fig. 1). However, follow-up was terminated after three consecutive negative cultures for NTS. All stool samples or rectal swabs were transported as fecal swabs in Cary-Blair Transport Medium to the Microbiology Laboratory in KEMRI in Nairobi. The stool samples were processed and suspected *Salmonella* isolates were identified as previously described (1), through biochemical characterization using API 20E and serotyping.

Data were electronically captured through the mobile-based Epicollect data collection tool (28). Descriptive statistics are presented as counts, proportions, and/or percentages.

### Whole-genome sequencing and analysis

Genomic DNA was extracted from bacterial overnight cultures using Quick-DNA Fungal/Bacterial Kits (Zymo Research) and quantified using qubit. Sequencing libraries were prepared following the Nextera XT prep kit (Illumina, Inc), and sequenced on Illumina NextSeq 2000 using a P3 cartridge, at the Genome Competence Centre, Robert Koch Institute in Berlin with an output of 2×300 bp paired end reads. Raw reads were trimmed for low-quality bases using fastp (29) and Kraken (30) used to determine the preliminary taxonomic classification of the isolates. Trimmed reads were then uploaded onto Enterobase (https://enterobase.warwick.ac.uk/) for draft genome assembly, serotype prediction using SeqSero2 (31), and multi-locus sequence typing (MLST) based on the 7 loci MLST scheme for *Salmonella* (Achtman 7 Gene MLST). GrapeTree (32) web-server tool was employed to visualize the genomic clustering patterns of NTS. In addition, the trimmed reads were subjected to *de novo* assembly using SPAdes version 1.1.0 (33). Thereafter, whole-genome mapping was performed using Bwa (34) and multi-sample variant calling was done using freebayes (35). Mapping reference was GCF_000006945.2 for *S*. Typhimurium and GCF_000009505.1 for *S*. Enteritidis. The phylogenetic tree was constructed using phyML (36) and the resulting trees were visualized through iTOL version 6.9.1 (37).

## RESULTS

### Recruited participants and prevalence of NTS

The total number of participants recruited from the four outpatient health facilities was 3,293 (Table 1), with most of the children being male (53.05%, 1,747/3,293). The majority (55.6%, 1,831/3,293) of the recruited children were aged ≤2 years.

**TABLE 1:** Distribution of recruited and NTS-positive participants per age

	Recruited participants			NTS positive cases		
Age group	Male	Female	Total	Male	Female	Total (%)
≤12 months	474	393	867	12	3	15 (1.73)
13–24 months	526	438	964	9	7	16 (1.66)
25–36 months	303	269	572	3	3	6 (1.05)
37–48 months	239	252	491	1	3	4 (0.81)
49–60 months	205	194	399	6	3	9 (2.26)
Total	1,747	1,546	3,293	31	19	50 (1.52)

The prevalence of nontyphoidal *Salmonella* (*S*. Typhimurium, *S*. Enteritidis, and other NTS serotypes) among the 3,293 children recruited, was 1.52% (index cases = 50) (Table 1). The majority of the NTS index cases were male (62%, 31/50); however, the proportion of male index cases (1.77%, 31/1,747) was comparable to the female (1.23%, 19/1,546). NTS positivity rate per age group was highest among children aged 49–60 months (2.26%) and lowest among the age group 37–48 months (0.81%) (Table 1). The highest proportion of positive cases was among males aged 49–60 months (2.93%, 6/205). Among the index cases, 94% (47/50) had NTS-positive stool samples, 4% (2/50) had NTS-positive blood samples and from one child, *S*. Enteritidis was isolated from both blood and stool. Two of the three cases with bacteremia were female, in addition, the two were also positive for *S*. Enteritidis. Among the 50 index cases, 25 (50%) were positive for *S*. Enteritidis, 18 (36%) for *S*. Typhimurium, 2 for *S*. Heidelberg, 2 for *S*. Saintpaul, and 3 cases positive for *S*. Kiambu, *S*. Eastbourne, and *S*. Newport, respectively. All the *S*. Enteritidis strains in this study, including the three recovered from blood samples, were of ST11, while all *S*. Typhimurium isolates except one of an unknown ST belonged to ST19. None of the invasive NTS cases was positive for *S*. Typhimurium or belonged to *S.* Typhimurium ST313.

### Persistence and shedding of NTS among index cases post-acute disease

**Fig 2:**
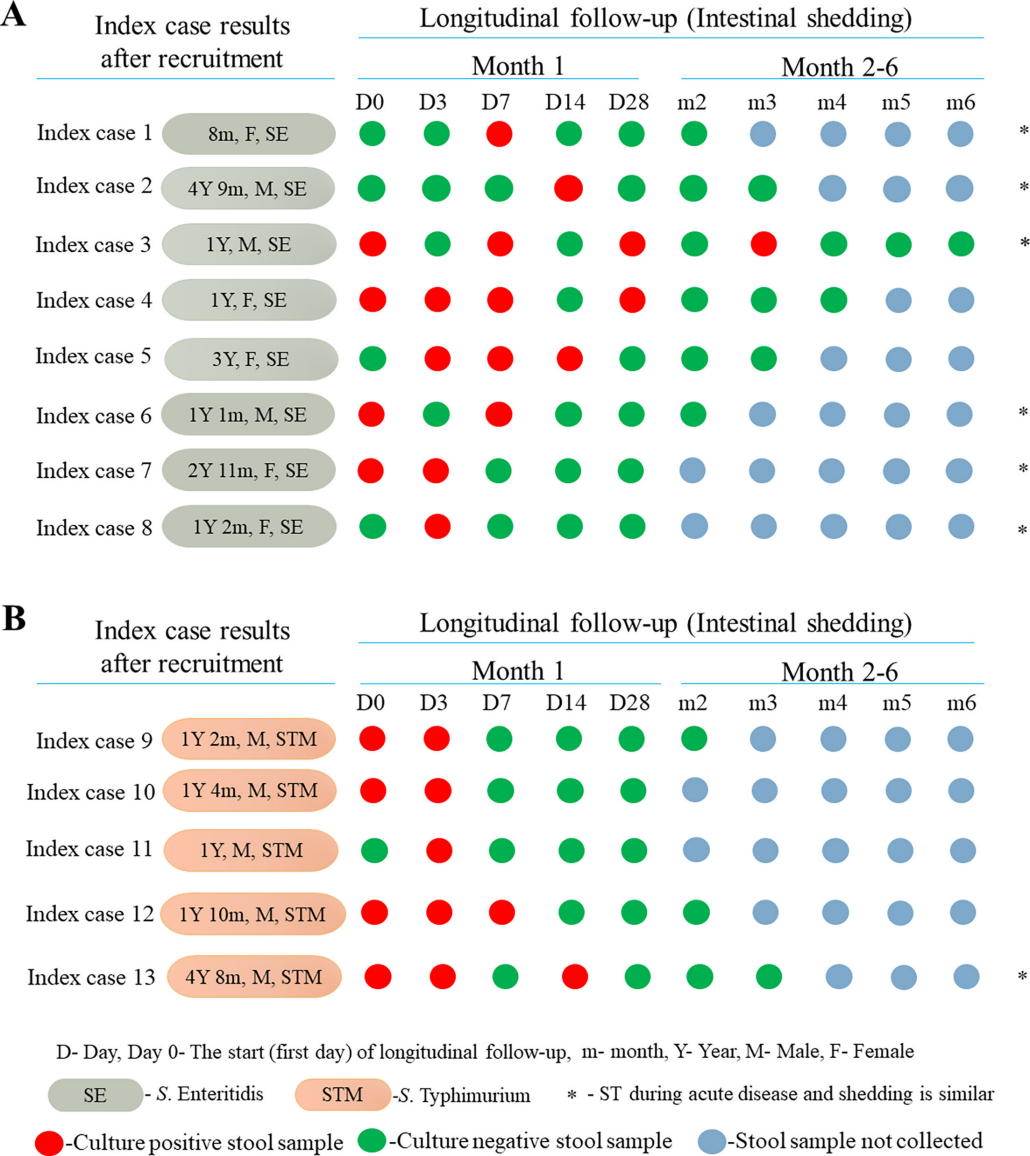
Asymptomatic shedding during longitudinal follow-up of index cases post-treatment. Intestinal shedding pattern of index cases who were positive for (**A**) *S.* Enteritidis and (**B**) *S.* Typhimurium during acute disease. Stool samples were collected during longitudinal follow-up and subjected to culture. Day 0 (D0) is the start (first day) of follow-up. Follow-up was stopped after three consecutive negative cultures. *From index cases 1, 2, 3, 6, 7, 8, and 13, the NTS STs (ST11 and ST19) recovered during acute disease were also recovered from faecal samples throughout the follow-up period.

Household follow-up of the index cases was done 14 days after completion of treatment. The follow-up rate among the cases was 84% (42/50). Among the eight index cases who were not followed up, seven either declined follow-up or moved out of the study area, while one died during admission for severe pneumonia. Among the 42 index cases followed-up antibiotic prescription was as follows: co-trimoxazole was prescribed to 18 index cases, amoxiclav to 16 index cases, and 4 index cases received cefuroxime, while another 4 index cases were each prescribed either azithromycin, doxycycline, erythromycin, and amoxicillin. The average duration of medication was 8 days, with the antibiotics being administered orally. Asymptomatic shedding (Fig. 2) was observed in almost one-third (31%, 13/42) of the index cases who were followed up post-treatment with a majority (69.2%, 9/13) of those being male. Among the index cases found to be shedding, majority (53.8%, 7/13) had received cotrimoxazole, three index cases were treated with amoxiclav, two were treated with cefuroxime, while one was treated with erythromycin.

The longest duration of shedding was 3 months post-treatment (Fig. 2A), which was observed in a 12-month-old male (index case 3). *S*. Enteritidis ST11 which was isolated during acute disease was also isolated during the 3 months of intermittent shedding. Similar findings were observed in index cases 1, 2, 6, 7, and 8 from whom *S*. Enteritidis ST11 was recovered during acute disease and shedding (Table S1). One of the three index cases (index case 8) with bacteremia during acute disease was found to be shedding the same serotype (*S*. Enteritidis ST11) during the second follow-up (D3), however, during acute disease the fecal sample was culture-negative for NTS. Index case 13 was shedding *S*. Typhimurium ST19 during follow-up, which was the same serotype and ST recovered during acute disease. However, of the 13 index cases observed to be shedding NTS post-treatment, 2 (index cases 10 and 11) were found to be shedding a different serotype compared to the one recovered during acute disease (Table S1). Four index cases (index cases 4, 5, 9, and 12) were shedding either a different serotype or the same serotype as the one recovered during acute disease at different days of follow-up. Index case 4 was shedding *S*. Brandenburg ST249 during the first follow-up (D0), however, in subsequent follow-up (D3, D7, and D28), the case was shedding the same NTS ST (*S*. Enteritidis ST11) as the one recovered during acute disease. A similar observation was recorded in index case 5 who was shedding *S*. Braenderup ST22 and *S*. Typhimurium ST19 during the second and third follow-ups in contrast to the *S*. Enteritidis ST11 recovered during acute disease and the fourth follow-up. Index case 12 was shedding a different serotype (*S*. Braenderup ST22) during the second follow-up (D3) compared to *S*. Typhimurium ST19 recovered during acute disease, the first (D0) and third (D7) follow-ups (Table S1). Of the 29 isolates recovered from the 13 children during the follow-up period, 19 (65.5%) were *S*. Enteritidis, 7 were *S*. Typhimurium, 2 *S*. Braenderup, and 1 *S*. Brandenburg. The majority (96.6%, 28/29) of the NTS isolates recovered during post-treatment shedding from index cases were isolated in the first month of follow-up (Fig. 2).

### Asymptomatic shedding of NTS among healthy individuals (household contacts and control households)

Household follow-up and sampling was done five times in the first month. However, after the first month, in individuals with NTS culture-positive stool samples, follow-up was terminated after three consecutive negative samples. From the 42 case-contact households, 104 contacts were recruited: 13 children under 5 years of age and 91 individuals above 5 years (Table 2). A majority (62/104, 59.6%) of the recruited contacts were female. Among the 104 contacts, three individuals, all female were found to be shedding either *S*. Enteritidis ST11, *S*. Braenderup ST22 or *S*. Orion ST639.

**TABLE 2:** Distribution of recruited contacts and controls and NTS carriers

	Recruited			NTS carriers		
		Males	Females	Males	Females	Total
Contacts	≤5 Years	4	9	0	1	1
	˃5 Years	38	53	0	2	2
	Total	42	62	0	3	3
Control households	≤5 Years	22	32	1	3	4
	˃5 Years	29	54	0	1	1
	Total	51	86	1	4	5

^*a*^ Data are presented as numbers unless otherwise stated. The median age for contacts ≤5 years was 3 years with the age range being 8 months to 5 years. Among contacts >5 years, the median age was 28 years with the age range of 6–45 years. The median age for controls ≤5 years was 2 years 8 months with age ranging from 4 months to 5 years while among controls above 5 years, the median age was 24 years 3 months with age ranging from 5 years 6 months to 42 years.

From the control households, 137 individuals were recruited with the majority (63.7%, 86/137) being female. Among the recruited controls, 54 were children under 5 years, with 4 (7.41%) of them being NTS carriers, and 83 individuals were above 5 years (Table 2) and one of them was found to be shedding NTS. All the 241 contacts and controls recruited were followed up successfully, and 8 (3.3%) had asymptomatic NTS shedding. Among the eight asymptomatic carriers, seven (87.5%) were female and 37.5% (3/8) were shedding *S*. Enteritidis ST11. None of the eight individuals had more than one episode of shedding. All the five controls shedding NTS were from different households.

### Relatedness of NTS serotypes among index cases and asymptomatic carriers

Phylogenetic analysis was used to determine the relatedness of isolates obtained during disease and shedding among cases and asymptomatic carriers. From three case-contact households, three contacts were shedding NTS; however, the strains recovered were different from those isolated from the index cases in their respective households. However, among the five controls who were shedding NTS, three had the same serotypes (two *S*. Enteritidis ST11 and one *S*. Typhimurium ST19) as the ones isolated from their matching index cases (Table S1). The controls were recruited from different households 100 m away from the index case household.

**Fig 3:**
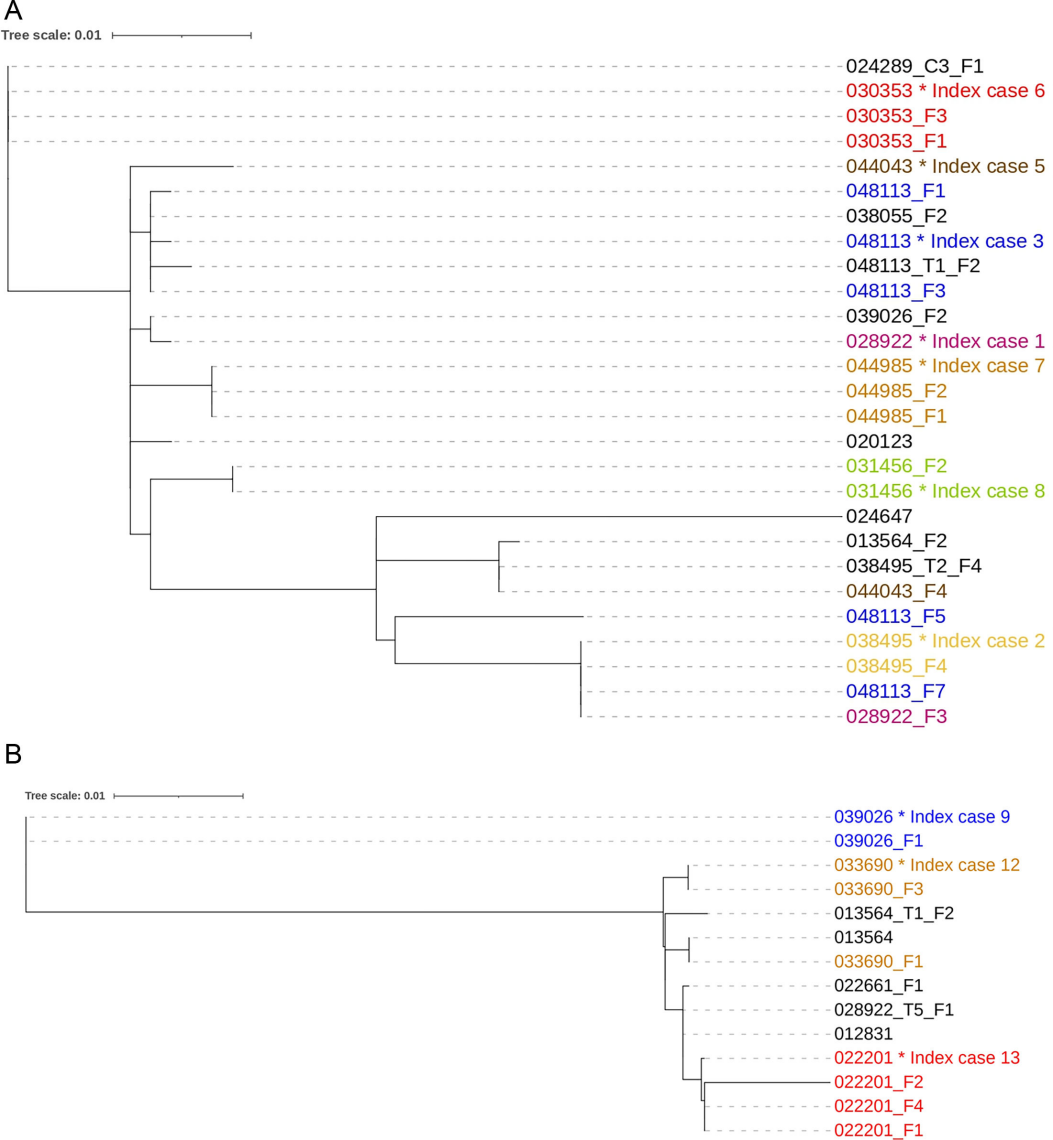
Phylogenetic tree of (**A**) 27 *S.* Enteritidis and (**B**) 14 *S.* Typhimurium isolates recovered during acute disease and post-treatment of index cases and asymptomatic carriers. The numbers/labels at the tip of tree are the participant and isolates identification. The numbers with the same color indicate isolates obtained from the same participant. * denotes the first isolate recovered from the index case during acute disease. F1 is the isolate recovered on the first day (D0) of follow-up. F2 is the isolate obtained on the second day (D3) of follow-up. F3 is isolate obtained on the third follow-up (D7), F4 is the isolate obtained on the fourth follow-up (D14), F5 is the isolate obtained on the fifth follow-up (D28), and F7 is isolate obtained on the seventh follow-up (M3). T1_F2 is the isolate obtained from the first control (healthy individual) on the second day of follow-up, T2_F4 is the isolate obtained from the second control during the fourth follow-up and T5_F1 is the isolate obtained from the fifth control during the first follow-up, C3_F1 is the isolate obtained from the third contact of an index case during the first follow-up.

In some of the index cases that were shedding NTS post-treatment, the isolates recovered during acute disease were closely related to subsequent isolates recovered during shedding thus forming a monophyletic group (Fig. 3). The phylogenetic distance between the isolates from index case 7 during acute disease and post-treatment shedding was six SNPs difference. In addition, isolates recovered from index case 3 (048113) and a control (048113_T1_F2) recruited from a household within 100 m of the case showed high phylogenetic relatedness. (Fig. 3A). However, from some index cases (index cases 1, 3, and 5) isolates recovered during asymptomatic shedding were distantly related to isolates obtained during acute disease (Fig. 3A).

## DISCUSSION

NTS remains an important cause of self-limiting enterocolitis globally and an often life-threatening invasive disease in sub-Saharan Africa. In this study, the prevalence of NTS among children below 5 years of age presenting at health facilities was 1.52% which is in agreement with findings from a previous study conducted in the same settings which reported a prevalence of 1.3% (27). Although the prevalence observed in both studies was low, these findings indicate that the disease remains endemic in these settings. However, other studies (38) have reported a higher prevalence of NTS among children under five in rural Kenya compared to urban settings with a recent study reporting a 5.3% prevalence (10). The difference in prevalence, in particular invasive disease, has been attributed to the holoendemicity of malaria in rural Kenya (39).

A high proportion of positive cases was observed among males aged 49–60 months. This could be an indication that children of this age group in particular males may be either exposed more to NTS among children under 5 years or have higher susceptibility. A study conducted in the United States reported that boys had a higher incidence of Salmonellosis although the difference was not statistically significant, and as mentioned in countries like the United States, NTS usually causes a different disease entity, namely self-limiting diarrhea (40).

In this study, children aged ≤2 years had a marginally higher proportion of NTS (1.67%) compared to those above 2 years (1.36%). Findings from previous studies conducted in Malawi (41), South Africa (42), four African sites and South Asia (10, 43), Tanzania (44), and Ghana (45) have shown that the prevalence of NTS infections is often higher in children below 2 years. These findings suggest that this age group is more likely to be exposed or could be more susceptible to NTS, which could infer that any future vaccine use may need to target children aged below 2 years. Several studies (1, 8, 10, 12, 22) have reported that *S*. Typhimurium is the predominant cause of bloodstream NTS infection in children; however, in the current study we observed that in the three children with iNTS, the serovar implicated was *S*. Enteritidis ST11. In addition, in contrast to previous studies which reported *S*. Typhimurium as the predominant NTS serotype, in this study *S*. Enteritidis ST11 was responsible for most of the NTS disease (50%) among index cases. Of interest is that in the present study, *S*. Typhimurium ST313 which has previously been implicated in iNTS (1, 22) within this setting was not observed which could be an indication of the changing epidemiology of iNTS in this area.

According to Buchwald et al. (46), the median duration of NTS excretion following an infection was ~5 weeks. However, other studies have reported that NTS shedding was eliminated 12 days post-initial detection in adults (47, 48). In the current study, we observed that 31% of index cases were shedding NTS with the longest duration being 3 months post-acute disease. This agrees with previous reports (15) which found that the persistence of NTS in humans could be between 30 days and 8.3 years. Buchwald et al. (46) reported that children below 5 years had a prolonged shedding period compared to adults; however, we observed in this case that the longest duration was 3 months. The 3-month duration is longer than the duration observed (21–28 days) in adults by Sirinavin et al. (16). The detection of phylogenetically related isolates from index cases during acute disease and post-treatment is an indication of NTS carriage, especially among children. However, the detection of other serotypes or distantly related isolates during asymptomatic shedding among index cases is an indication of either coinfection or a new infection event, which is similar to previous findings by Sirinavin et al. (16).

Although NTS is considered a zoonotic pathogen, especially in developed economies several studies have hypothesized that human reservoirs play a critical role in the transmission of NTS in sub-Saharan Africa (1, 8, 12). In this study, we found that in three households, the asymptomatic contacts were shedding different serotypes compared to those recovered from the index cases. This is in contrast to findings from a study (20) conducted in Malawi, which reported that in two households, the *S*. Typhimurium isolates from the index cases were closely related to the isolates from asymptomatic household members. However, we also observed that among the healthy individuals (controls) residing within 100 m of the index case households, three were shedding the same ST as those recovered from index cases during acute disease. One of the controls was shedding an isolate that was highly related to the one recovered from the index case during acute disease (Fig. 3A). The possibility of NTS transmission in households and communities is important to note as this could be a source of infection for vulnerable populations. This also highlights the plausible role of poor sanitation and hygiene in pathogen transmission in these low-resource settings. Considering that this study was undertaken in an urban informal settlement which is characterized by inadequate access to clean water, inadequate latrines, non-existent sewerage infrastructure, open drainage, and poor waste management, these factors could contribute to the transmission of the pathogens within households and the community.

The presence of healthy individuals shedding closely related NTS serotypes as the convalescing index cases, especially within the same location is an indication that asymptomatic carriers are possibly contributing to sustaining transmission of the pathogen within the community. Several studies in sub-Saharan Africa have also reported on the presence of shedding among asymptomatic hosts within the community setting (1, 12, 19). While the current study did not investigate the presence of NTS in the gallbladder of asymptomatic carriers, a recent case report showed that *S*. Enteritidis infection led to gallbladder empyema in a 52-year-old female patient (49). In this study we found a 40-year-old female who was shedding *S*. Enteritidis; however, the shedding was only observed once. It is important to note that, compared to *S*. Typhi, it is not common for NTS to form biofilms in gallstones which leads to carriage in the gallbladder. However, recent mouse model studies (50) have shown that NTS strains can form biofilms on gallstones and colonize the mouse gallbladder.

While this study reports on the duration and pattern of NTS shedding among children under 5 years post-convalescence, there are several limitations. The study could not establish whether the asymptomatic shedding in index cases was due to reinfection or coinfection events. Second, we did not investigate whether there was a common environmental reservoir or contaminated food that could have resulted in concurrent infection between index cases and asymptomatic carriers. Third, our first point of contact was the index case; thereafter, we sampled contacts and controls, therefore, we could not establish if the NTS infected the index case or the asymptomatic carriers first. However, of importance is that this study highlights the shedding patterns and the plausible role of NTS carriage in disease-endemic low-resource settings.

### Conclusion

This study observed that asymptomatic shedding of NTS in patients with diarrhogenic NTS disease can last for 3 months among children post-convalescence. Asymptomatic shedding of NTS was also observed in healthy individuals living close to the cases. The presence of asymptomatic carriers in low-resource settings is likely to play a role in human-to-human transmission in households and communities. These findings highlight the need to consider the introduction of vaccines, especially within the first 2 years of life to reduce the burden and severity of disease, but in the long-term improvement of water sanitation and hygiene infrastructure in low-resource settings will play an important role in prevention and control of NTS. There is needed to investigate the immunological factors associated with carriage, the seroprevalence of NTS in both index cases and controls during and after follow-up, the concentration of NTS in stool samples during carriage that would constitute an infectious dose among children and adults. Further, there is need to investigate the role of microbiome in disease and carriage and the rates of co-infection and reinfection among individuals in disease-endemic settings.

## Data Availability

Whole-genome sequence data were submitted to the National Center for Biotechnology Information Data Libraries (GenBank) under the BioProject ID PRJNA1153302.

## SUPPLEMENTAL MATERIAL

Table S1 jcm.00750-24-s0001.xlsx Serotypes and sequence types of nontyphoidal *Salmonella* recovered from cases and healthy individuals.

ASM does not own the copyrights to Supplemental Material that may be linked to, or accessed through, an article. The authors have granted ASM a non-exclusive, world-wide license to publish the Supplemental Material files. Please contact the corresponding author directly for reuse.
